# Leptin improves the *in vitro* development of preimplantation rabbit embryos under oxidative stress of cryopreservation

**DOI:** 10.1371/journal.pone.0246307

**Published:** 2021-02-02

**Authors:** Tarek A. Alshaheen, Mohamed H. H. Awaad, Gamal M. K. Mehaisen

**Affiliations:** 1 Department of Animal and Fish Production, College of Agricultural and Food Sciences, King Faisal University, Al-Ahsa, Saudi Arabia; 2 Department of Poultry Diseases, Faculty of Veterinary Medicine, Cairo University, Giza, Egypt; 3 Department of Animal Production, Faculty of Agriculture, Cairo University, Giza, Egypt; Friedrich-Loeffler-Institute, GERMANY

## Abstract

Vitrification is an economically effective method for embryo cryopreservation in human and livestock animals; however, it carries the risk of damage by the exposure to severe oxidative stress. The present study was conducted to evaluate the effect of leptin at different levels on the *in vitro* development of fresh and vitrified preimplantation embryos in a rabbit model. Normal embryos at morulae stage were randomly cultured for 2 h with 0, 10, 20 or 100 ng/mL of leptin, then were cultured for further 48 h as freshly or after vitrification. Thereafter, developed blastocysts form the best leptin level in fresh and vitrified embryos along with their controls were allocated to analyze the pro-oxidant (malondialdehyde, MDA; nitric oxide, NO), antioxidant (total antioxidant capacity, TAC; superoxide dismutase, SOD; glutathione peroxidase, GPx), apoptotic (Bcl-2 associated X protein, BAX; heat shock 60kD protein member 1, HSP60; tumor necrosis factor alpha, TNFα) and developmental (sex determining region Y box protein 2, SOX2; Nanog homeobox protein, NANOG; Octamer-binding protein 4, OCT4) biomarkers. Results indicate that expanding and hatching rates of embryos were significantly higher at 20 ng/mL leptin than the other levels, while vitrification had an independent suppression effect on the *in vitro* development rates. The MDA and NO were significantly higher, while TAC, SOD and GPx were significantly lower in the vitrified than fresh embryos. In contrast, leptin treatment significantly decreased the pro-oxidant biomarkers and increased the antioxidant biomarkers in both fresh and vitrified embryos. Vitrification significantly increased the antiapoptotic biomarkers, and decreased the developmental biomarkers in embryos. In contrast, leptin decreased the BAX and TNFα, increased the HSP60, and moreover, ameliorated the reduction of developmental biomarkers in the vitrified embryos. These results conclude that leptin could be used as antiapoptotic and antioxidant promotor to support the *in vitro* embryonic development, particularly under oxidative stress emerged from cryopreservation programs.

## Introduction

Embryo cryopreservation is widely used as an assisted reproductive technology for the preservation, reconstitution and distribution of valuable and/or endangered livestock populations [1,2]. Vitrification is an economically and effective simple method for embryo cryopreservation avoiding the mechanical damage of ice crystals [3,4]. In rabbits, embryo vitrification techniques have been studied and developed using various cryoprotectant solutions, carriers and devices, and also resulting in various *in vitro* development and *in vivo* survival rates [5,6]. However, the safe routine application of embryo vitrification may remain at a great challenge due to the risk of damage by exposure to suboptimal conditions during *ex vivo* manipulation and embryo cryopreservation per se [6–9].

Previous works has demonstrated that *in vitro* produced and vitrified embryos could be injured by the exposure to oxidative stress through induction of membrane lipid peroxidation, protein oxidation, cellular anti-ROS-defense suppression, metabolism disruption, mitochondrial dysfunction, cell death and apoptosis [7,10–14]. It was found that vitrification suppressed the expression of cellular antioxidant enzyme system such as superoxide dismutase (SOD), glutathione-s-transferase (GST) and glutathione peroxidase (GPx) [7,15]. Recent studies reported that vitrification and oxidative stress negatively affected the embryo expression of several proteins encoded by developmentally-related-genes such as gap junction protein alpha 1 (GJA1), nanog homeobox protein (NANOG) and octamer-binding protein 4 (OCT4) [7,15,16]. Moreover, other reports correlated the low developmental competence of embryos to the presence of some pro-oxidant proteins such as malondialdehyde (MDA) and nitric oxide (NO) [7] and some apoptotic proteins like Bcl-2 associated X protein (BAX), heat shock 60kD protein member 1 (HSP60) and tumor necrosis factor alpha (TNFα) [17,18].

Leptin is an obese (*ob*) gene encoded hormone mainly secreted by adipocyte cells and has a wide role in the regulation of body weight, obesity, appetite and energy balance [19,20]. There is much evidence that leptin also serves as a modulator of steroidogenesis, sexual maturation, fertility and other reproductive functions [21–25]. In addition, leptin and its receptors have been expressed in the granulosa, cumulus and luteal cells, oocytes and embryos of various animals such as buffalo [26,27], bovine [28,29], porcine [30,31] and mouse [20,32], and in human [33]. In this context, Kawamura et al. [20] found that culture of mouse preimplantation embryos with 100 ng/mL leptin increased the proliferation of the inner cell mass (ICM) and trophectoderm (TE) cells and promoted the development of embryos to expanded and hatched blastocysts. In contrast, Herrid et al. [34] found the same beneficial effect of leptin at 10 and 100 ng/mL levels on embryo development in mouse and sheep species but based on the leptin concentration and embryonic developmental stage. More recently, Kšinanová et al. [32] suggested that leptin promotes survival rather than apoptosis when it was supplemented *in vitro* at levels of 10 ng/mL to preimplantation embryos recovered from obese or normal mouse females. Furthermore, Panda et al. [27] reported that leptin supplementation at 10 ng/mL to oocytes and zygotes of buffalo increased their blastocyst rate accompanying with upregulation of some developmental genes such as OCT4, hyaluronidase synthetase 2 (HAS2), epidermal growth factor receptor (EGFR) and leptin receptor (LEPR).

Although leptin could be used to promote the *in vitro* development of embryos, the hypothesis that leptin can protect preimplantation embryos against the deleterious effects of oxidative stress during *in vitro* culture and cryopreservation has not been reported in the literatures. Therefore, the current study was conducted to evaluate the effect of leptin at different levels on the *in vitro* development of fresh and vitrified preimplantation embryos in a rabbit model. In addition, the effect of leptin on the expression of some pro-oxidant, antioxidant, apoptotic and developmental biomarkers were further assessed in the fresh and vitrified rabbit embryos.

## Materials and methods

### Animals and ethical statement

Fifty nulliparous, 4.5–5 m old and 2.5–3.0 kg BW, New Zealand White rabbit does and 10 bucks were obtained from the Station of Agricultural Experiments, Faculty of Agriculture, Cairo University for the current work. During the experimental period, all rabbits were housed individually in cages settled in a semi-closed pen system, and supplied with galvanized-steel feeders and automatic drinking nipples. Rabbits were kept in identical environmental conditions (light alternating on a cycle of 16L:8D h and temperature range of 16–20 °C) and provided with identical commercial diet (18.4% crude protein, 3.1% ether extracts, 12.7% crude fiber, 8.5% ash and 1.26 MJ/kg metabolizable energy) and free access to water.

All experimental procedures in the present study were reviewed and approved by the ethical board of Cairo University-Institutional Animal Care and Use Committee (CU-IACUC). The approval number for this study is: CU-II-F-14-19. The method of sacrificing animals in the present study was applied by slitting the neck for cutting the carotid arteries, jugular veins, esophagus and trachea, without severing the spinal cord or the head, while the animal is alive.

### Embryo collection, treatments and experimental design

**Fig 1:**
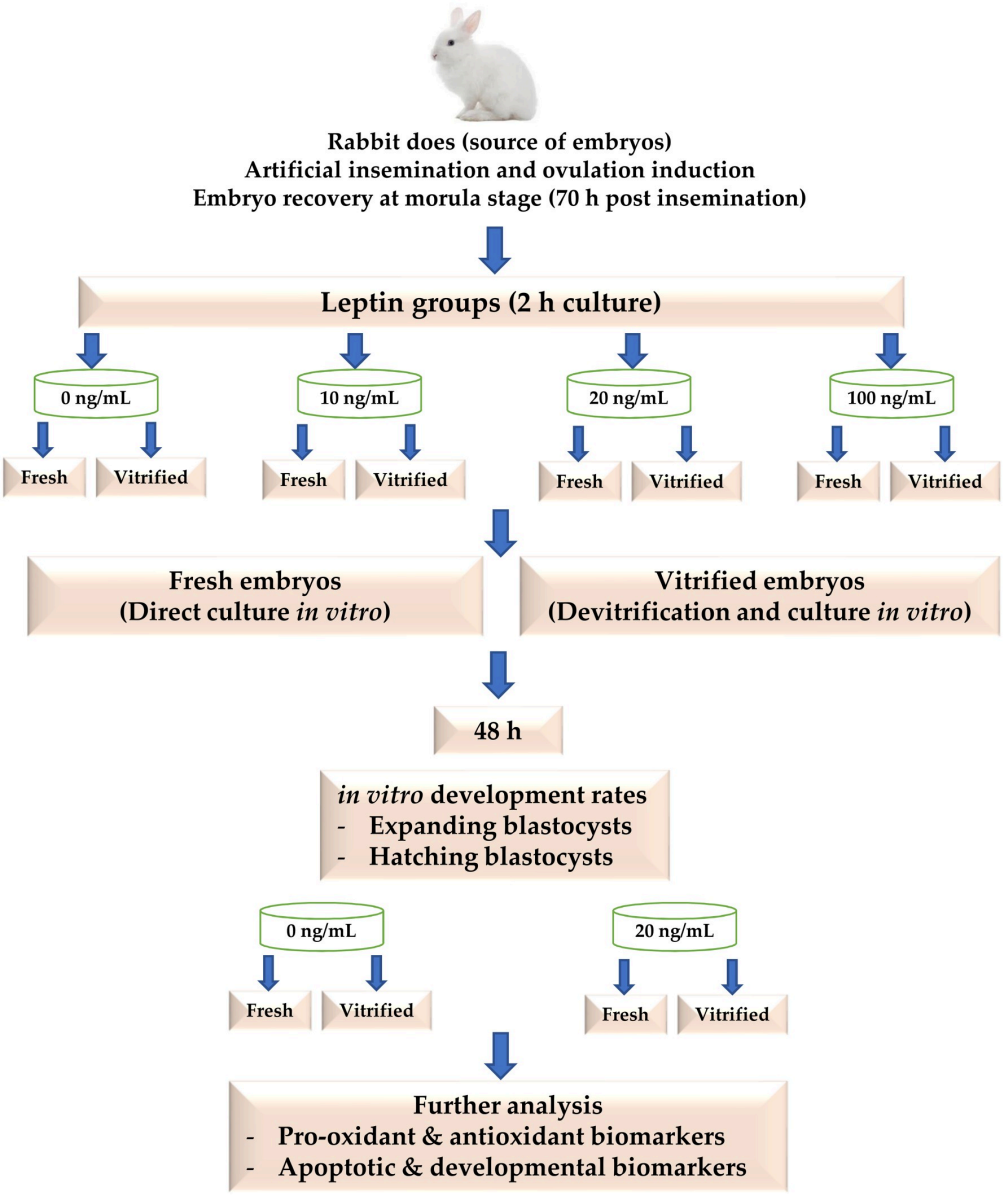
A schematic diagram of the experimental procedures.

Rabbit does were stimulated for receptivity by an i.m. injection of 40 IU eCG (Gonaser, HIPRA Laboratories, S.A., Girona, Spain) 60 h before insemination. Each rabbit doe was artificially inseminated with 0.5 ml of semen pools previously recovered from the bucks and assessed for at least more than 70% motility, less than 15% abnormality and a concentration of approximately 10x10^6^ sperm/mL; according to the methods described by [35]. Immediately after insemination, rabbit does were induced for ovulation by an i.m. injection with 0.2 mL solution that contained 0.8 μg buserelin acetate (Receptal, MSD Animal Health, New Cairo, Egypt). At 70 h post insemination, rabbit does were sacrificed and embryos were collected from uterine horns at room temperature by using flushing medium [1 L Dulbecco’s phosphate-buffered saline (DPBS, Sigma-Aldrich Chemicals S.A., Egypt) containing 0.132 g calcium chloride, 0.2% bovine serum albumin (BSA, Sigma) and 0.1% antibiotics (10,000 units penicillin-G and 10 mg streptomycin per ml; penicillin–streptomycin solution 100X, BioShop Canada Inc.)]. Only the normal embryos (compact morula with intact mucin coat and zona pellucida) obtained from each rabbit doe were randomly distributed in 4-well embryo culture dishes (Nunc A/S, Thermo Fisher Scientific, Roskilde Site, Denmark), 10–15 embryos per well. Each well contained 1 mL of tissue culture medium (TCM 199, Sigma), provided with 20% fetal calf serum (Sigma) and 0.1% antibiotics, and supplemented with either 0, 10, 20 or 100 ng/mL of recombinant rabbit leptin (#CYT507, BioShop Canada Inc.). Embryos were cultured with leptin for 2 h at 38.5°C, 5% CO2 and saturated humidity. After that, embryos of each leptin level were either transferred directly to fresh culture media (Fresh embryos) or cultured after implementation of a vitrification/devitrification protocol (Vitrified embryos) as described below. After 48 h of embryo culture, the in vitro development rates to expanded and hatched blastocyst stages were recorded. Thereafter, the developed blastocysts form the best leptin level in fresh and vitrified embryos along with their controls (0 ng/mL) were harvested for further analysis of pro-oxidant, antioxidant, apoptotic and developmental biomarkers as mentioned below. A schematic diagram of the experimental procedures is shown in Fig 1.

### Vitrification/Devitrification protocol

Embryos were vitrified in two steps according to the protocol described in a previous work [36]. In brief, embryos were first plated for 2 min in a vitrification medium consisting of 12.5% (v/v) of both dimethyl sulphoxide (DMSO, Sigma) and ethylene glycol (EG, Sigma) added to flushing solution as mentioned above. Then embryos were plated for 1 min in a solution consisted of 20% (v/v) of both DMSO and EG in flushing solution before loading into 0.25 mL-volume plastic straws (IMV Technologies, L’Aigle, France). The straws were sealed and directly immersed into liquid nitrogen (LN_2_). Devitrification of embryos was performed immediately by taking out the straws from LN_2_ and immersing directly in water bath at 20°C for 10–15 s. The embryos were expelled from the straws into a plate containing 0.33 M sucrose (Sigma) dissolved in DPBS solution for 5 min, then washed in another plate containing DBPS solution only for another 5 min.

### *In vitro* development rates

All rabbit females in the present study were successfully ovulated and a total of 1089 embryos were recovered in 10 recovery sessions (5 females per session, two sessions at 3 days interval per week). In each recovery session, abnormal embryos were discarded and only normal embryos at morulae stage were randomly distributed for leptin and vitrification treatment groups and then cultured as previously stated. Nine hundred eighty one normal embryos in total were found suitable in all recovery sessions and they were randomly allocated into 135+155, 109+100, 137+138 and 104+105 as fresh + vitrified embryos for 0, 10, 20 and 100 ng/mL leptin groups, respectively. The fresh and vitrified/devitrified embryos in each leptin level were examined during culture period using stereo microscope at 7-45X magnification (ZTX-PW7045, Ningbo ProWay Optics & Electronics Co., Ltd., Zhejiang, China). After the first 24 h of incubation, the percentage of morula reached to expanded blastocyst stage was considered as the expanding rate of initial cultured embryos. After the second 24 h of incubation, the number of hatching and hatched blastocysts were recorded and calculated as the hatching rates of initial cultured embryos, respectively. Images of the developmental stages obtained from cultured embryos in the current study are shown in S1 Fig.

### Pro-oxidant and antioxidant biomarkers assay

Based on the preliminary results obtained from the *in vitro* development rates, 60 hatched blastocysts (divided equally into 6 biological replicates) were harvested from either fresh or vitrified embryo groups in both leptin levels (0 and 20 ng/mL). Embryos in each replicate were homogenized according to the methods described in a recent work [15]. Briefly, embryos were subjected to freeze-thaw cycles to break down the embryo cells through washing three times with 1 mL phosphate-buffered saline (PBS, BioShop Canada Inc.) and collecting by centrifugation at 1030 g for 10 min at 4°C. The supernatant was then collected and the total protein was quantified on the principle of biuret colorimetric reaction [37], according to the kit’s protocol (MBS2540542, MyBioSource, Inc., San Diego, USA), in order to normalize all data to the protein concentration in each sample. After that, the malondialdehyde (MDA) and nitric oxide (NO) as pro-oxidant biomarkers, and the total antioxidant capacity (TAC), superoxide dismutase (SOD) and glutathione peroxidase (GPx) as antioxidant biomarkers were assayed in the supernatant. All determinations were performed following instructions of colorimetric assay kits ((MBS822354, MBS480450, MBS2540515, MBS841580 and MBS480417 for MDA, NO, TAC, SOD and GPx assays, respectively; MyBioSource, Inc., San Diego, USA), and the measurements were obtained using an automatic scanning spectrophotometer (Model-CE1010, Cecil Instruments Limited, Cambridge, United Kingdom). The average inter- and intra-assay coefficient of variation (CV) were 8.0% and 11.9% for MDA, 6.1% and 7.5% for NO, 5.6% and 8.3% for TAC, 12.1% and 9.2% for SOD, and 9.4% and 10.0% for GPx, respectively.

### Apoptotic and developmental biomarkers assay

As described above, the supernatant was also used for the assessment of some apoptotic biomarkers such as bcl-2 associated X protein (BAX), heat shock 60kD protein member 1 (HSP60) and tumor necrosis factor alpha (TNFα), and some developmental biomarkers such as sex determining region Y box protein 2 (SOX2), nanog homeobox protein (NANOG) and octamer-binding protein 4 (OCT4). These proteins were determined according to the manufacturer’s protocols using specific ELISA kits (MBS732916, MBS2707501, MBS700705, MBS2511080, MBS763634 and MBS2502128 for BAX, HSP60, TNFα, SOX2, NANOG and OCT4 assays, respectively; MyBioSource, Inc., San Diego, USA). The measurements were recorded by an automated ELISA (Model 550 Microplate Reader, Bio-Rad Laboratories Inc., USA), and data were normalized to the protein concentration in each sample. The average inter- and intra-assay CV for ELISA tests were 6.1% and 9.7% for BAX, 7.8% and 10.1% for HSP60, 5.8% and 7.1% for TNFα, 10.1% and 9.7% for SOX2, 8.4% and 8.5% for NANOG, and 6.0% and 8.5% for OCT4, respectively.

### Statistical analysis

A binary probit link with binomial error distribution was established in a generalized linear model to analyze the main effects of leptin levels (0, 10, 20 and 100 ng/mL), vitrification treatment (fresh ad vitrified embryos) and their interactions on the parameters of *in vitro* development rates. Data of pro-oxidant, antioxidant, apoptotic and developmental biomarkers were subjected to one-way ANOVA to analyze the differences between the 4 embryo groups (fresh embryos or vitrified embryos cultured without or with 20 ng/mL leptin). The significance of means was detected by Duncan’s multiple-range tests, and the significance level was set at *P <* 0.05. Results of *in vitro* development are expressed as means ± standard error (SE), while data of biomarkers are presented as means ± standard deviation (SD). All statistical analyses were performed using IBM-SPSS-22.0 Software Package (IBM corp., NY, USA, 2013).

## Results

### Embryo *in vitro* development rates

**Fig 2:**
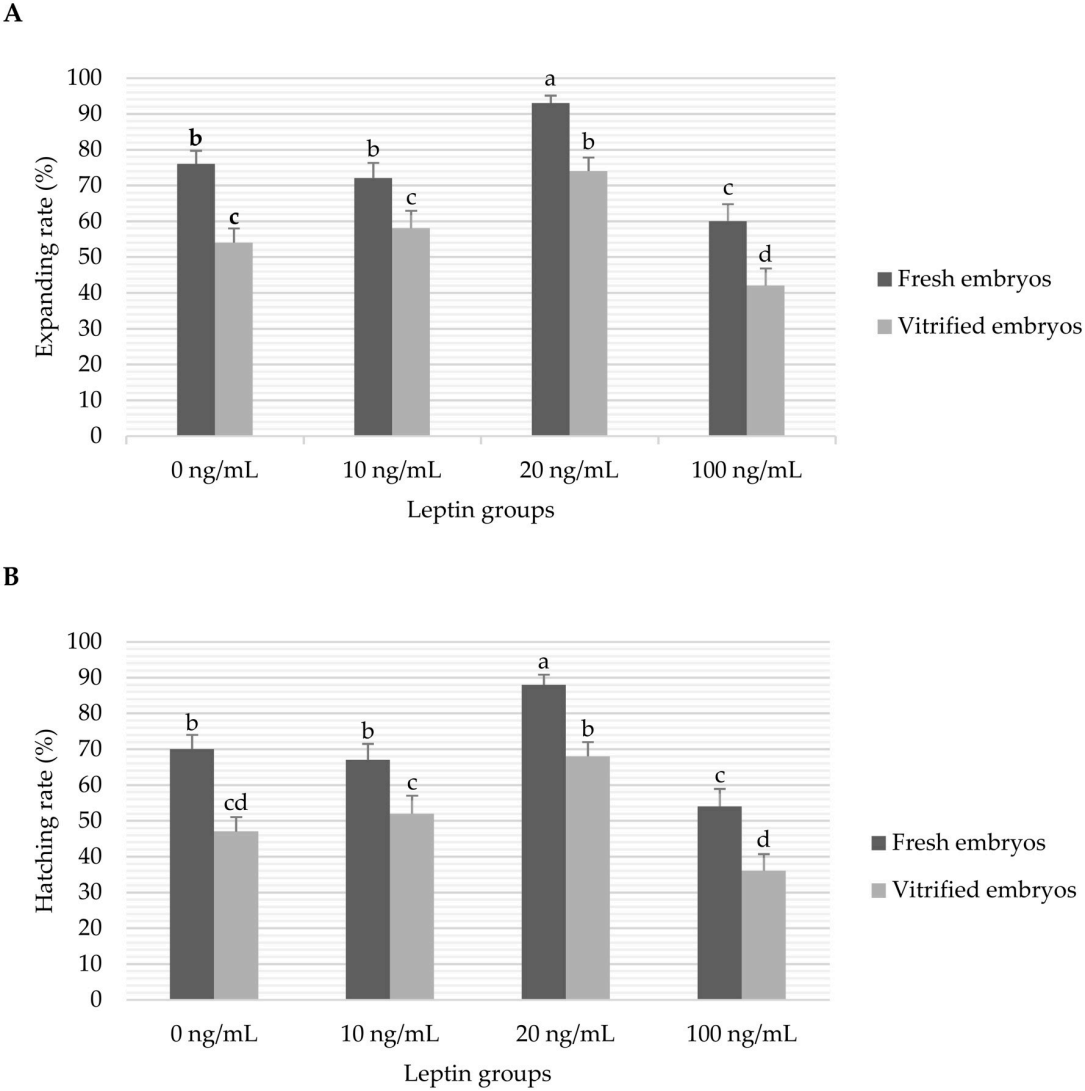
Data of *in vitro* expanding (A) and hatching (B) blastocyst rates of fresh and vitrified rabbit embryos cultured with different levels of leptin. Bars represent the mean ± standard error (SE) and the different superscripts (a, b, c, d) indicate the significant differences between means (P<*0*.*05*). Number of initial cultured embryos per fresh/vitrified treatment group were 135/155, 109/100, 137/138 and 104/105 embryos for 0, 10, 20 and 100 ng/mL leptin groups, respectively. Expanding and hatching rates were calculated as percentages of expanded and hatched blastocysts from the initial cultured morulae embryos, respectively, in each treatment group.

The overall data of *in vitro* developmental rates of fresh and vitrified embryos cultured with different levels of leptin are presented in Fig 2. There were no significant interactions between the leptin and vitrification treatments for the analysed variables of development. The expanding rates were significantly (*P* < 0.05) higher in the embryos cultured with 20 ng/mL leptin (93% and 74% in the fresh and vitrified embryos, respectively) than the other groups (76, 72 and 60% in the fresh embryos and 54, 58 and 42% in the vitrified embryos cultured with 0, 10 and 100 ng/mL leptin, respectively). In addition, the hatching rates were significantly (*P* < 0.05) higher in the embryos cultured with 20 ng/mL leptin (88% and 68% in the fresh and vitrified embryos, respectively) than the other groups (70, 67 and 54% in the fresh embryos and 47, 52 and 36% in the vitrified embryos cultured with 0, 10 and 100 ng/mL leptin, respectively). As shown in Fig 2, the vitrification treatment significantly (*P* < 0.05) decreased the expanding and hatching rates of embryos in all leptin groups.

### Pro-oxidant and antioxidant biomarkers

**Fig 3:**
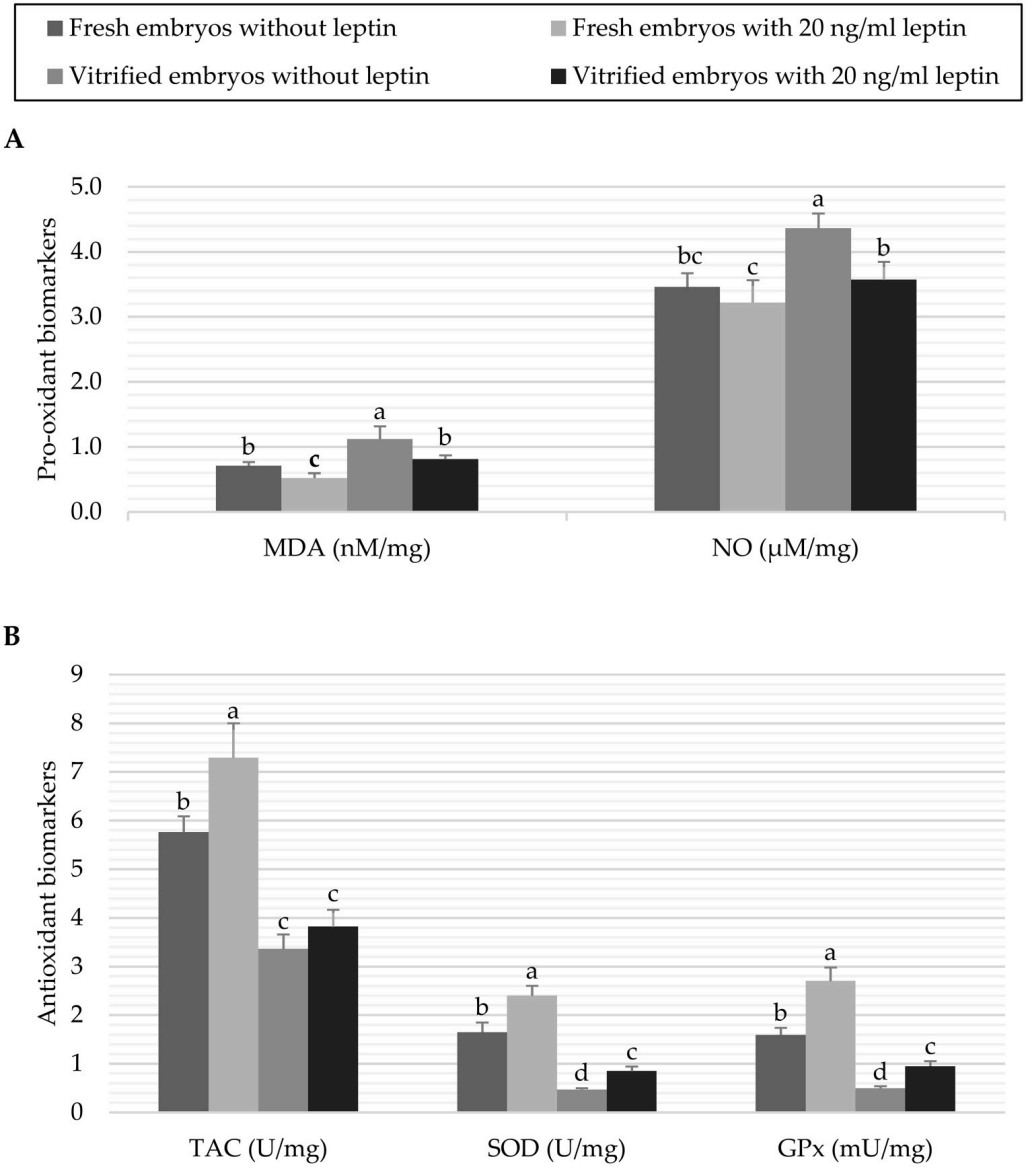
Data of pro-oxidant biomarkers (A) and antioxidant biomarkers (B) of fresh or vitrified rabbit embryos cultured with or without leptin. Bars represent the mean as per mg of total protein ± standard deviation (n = 6). Bars with different letters (a, b, c, d) are significantly different (P<*0*.*05*). Pro-oxidant biomarkers: malondialdehyde (MDA), and nitric oxide (NO); Antioxidant biomarkers: total antioxidant capacity (TAC), superoxide dismutase (SOD), and glutathione peroxidase (GPx).

The results of pro-oxidant and antioxidant biomarkers in fresh and vitrified embryos treated with 0 or 20 ng/mL leptin are presented in Fig 3. It was observed that vitrification of embryos without leptin treatment significantly (*P* < 0.05) increased the pro-oxidant levels compared to their levels in the control fresh embryos (1.12±0.195 *vs*. 0.71±0.057 nM/mg MDA and 4.36±0.231 *vs*. 3.46±0.212 μM/mg NO, respectively). While culture of embryos with 20 ng/mL leptin decreased the pro-oxidant levels in both fresh (0.52±0.076 nM/mg MDA and 3.22±0.345 μM/mg NO) and vitrified (0.81±0.061 nM/mg MDA and 3.57±0.275 μM/mg NO) embryos, compared to their controls (Fig 3A). On the other hand, leptin significantly (*P* < 0.05) promoted the activation of antioxidant biomarkers in fresh embryos (7.29±0.708 *vs*. 5.76±0.326 U/mg TAC, 2.40±0.202 *vs*. 1.65±0.201 U/mg SOD and 2.70±0.278 *vs*. 1.59±0.150 mU/mg GPx in the embryos cultured with 20 ng/mL leptin *vs*. control embryos, respectively). In contrast, vitrification treatment significantly (*P* < 0.05) decreased the antioxidant biomarkers (3.36±0.297 or 3.82±0.345 U/mg TAC, 0.47±0.026 or 0.85±0.092 U/mg SOD and 0.49±0.047 or 0.95±0.102 mU/mg GPx in the vitrified embryos treated with 0 or 20 ng/mL leptin, respectively) compared to the fresh control embryos (Fig 3B).

### Apoptotic and developmental biomarkers

**Fig 4:**
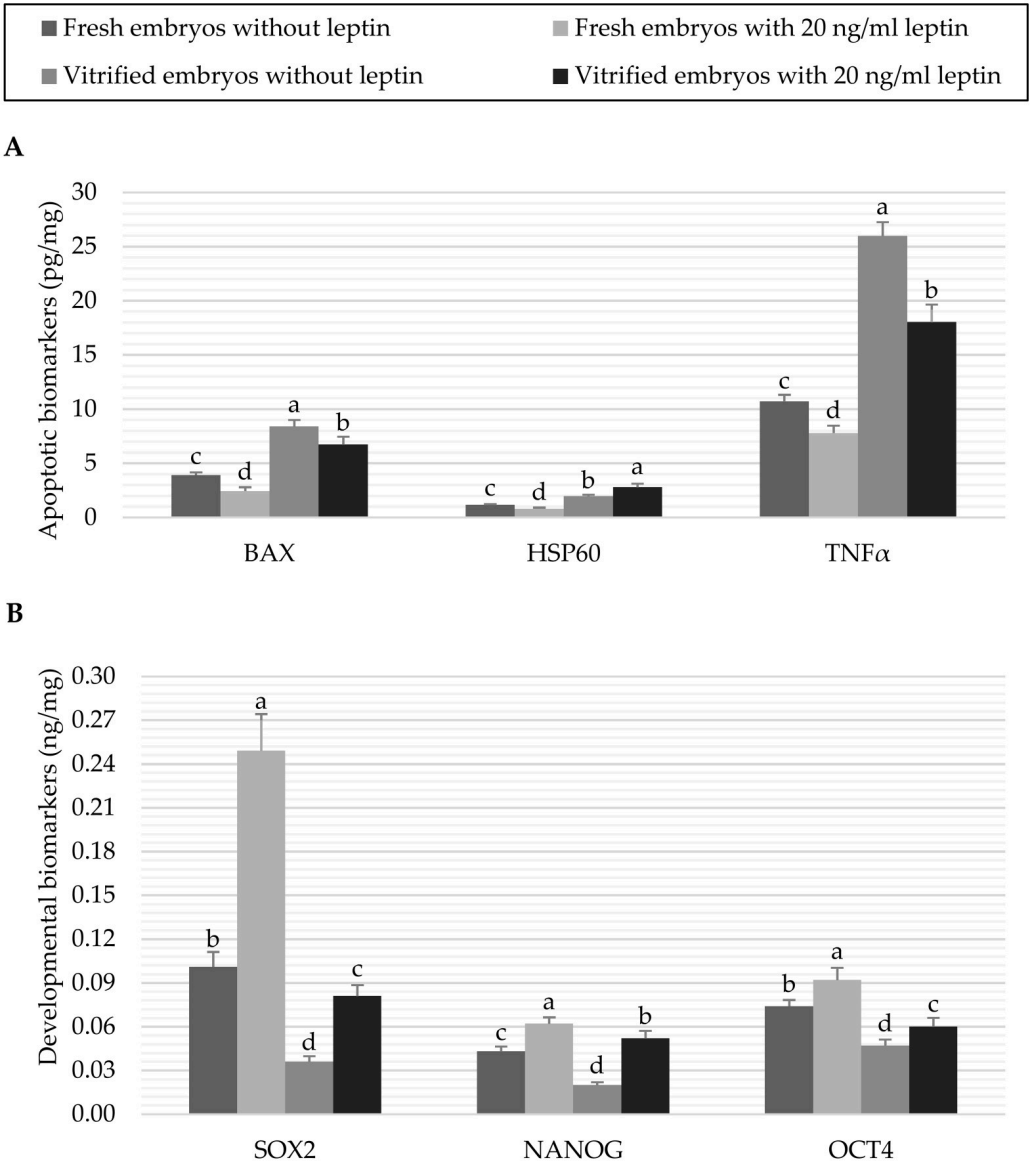
Data of apoptotic biomarkers (A) and developmental biomarkers (B) of fresh or vitrified rabbit embryos cultured with or without leptin. Bars represent the mean as per mg of total protein ± standard deviation (n = 6). Bars with different letters (a, b, c, d) are significantly different (P<*0*.*05*). Apoptotic biomarkers: Bcl-2 associated X protein (BAX), heat shock 60kD protein member 1 (HSP60), and tumor necrosis factor alpha (TNFα); Developmental biomarkers: Sex determining region Y box protein 2 (SOX2), Nanog homeobox protein (NANOG), and Octamer-binding protein 4 (OCT4).

The results of apoptotic and developmental biomarkers in fresh and vitrified embryos treated with 0 or 20 ng/mL leptin are shown in Fig 4. Vitrification of embryos significantly (*P* < 0.05) increased the BAX, HSP60 and TNFα apoptotic proteins in the embryos treated or not treated with leptin in comparison with the fresh control embryos (Fig 4A). In contrast, leptin treatment significantly (*P* < 0.05) decreased the levels of the apoptotic biomarkers in fresh embryos (2.43±0.358 *vs*. 3.91±0.239 pg/mg BAX, 0.80±0.110 *vs*. 1.15±0.089 pg/mg HSP60 and 7.79±0.683 *vs*. 10.70±0.619 pg/mg TNFα in embryos cultured with 20 *vs*. 0 ng/mL leptin, respectively). In vitrified embryos, culture with leptin significantly (*P* < 0.05) reduced the levels of BAX and TNFα which highly expressed by vitrification (6.73±0.713 *vs*. 8.39±0.608 pg/mg BAX and 18.02±1.619 *vs*. 25.96±1.281 pg/mg TNFα in embryos cultured with 20 *vs*. 0 ng/mL leptin, respectively), while HSP60 was significantly (*P* < 0.05) increased by leptin (2.78±0.327 *vs*. 1.95±0.146 pg/mg in embryos cultured with 20 *vs*. 0 ng/mL leptin). On the other hand, leptin significantly (*P* < 0.05) increased the expression of the examined developmental proteins in the fresh embryos (0.25±0.025 *vs*. 0.10±0.010 ng/mg SOX2, 0.06±0.004 *vs*. 0.04±0.003 ng/mg NANOG and 0.09±0.008 *vs*. 0.07±0.004 ng/mg OCT4 in embryos cultured with 20 *vs*. 0 ng/mL leptin, respectively). After vitrification, the developmental biomarkers were significantly (*P* < 0.05) decreased compared to the fresh control embryos; however, the previous culture with 20 ng/mL leptin ameliorated the reduction in these developmental biomarkers (0.08±0.008 *vs*. 0.04±0.004 ng/mg SOX2, 0.05±0.005 *vs*. 0.02±0.002 ng/mg NANOG and 0.06±0.006 *vs*. 0.05±0.004 ng/mg OCT4 in embryos cultured with 20 *vs*. 0 ng/mL leptin, respectively, Fig 4B).

## Discussion

Since approving the vitrification techniques as properly considerable methods for cryopreservation of mammalian embryos [38], many attempts have been made in the last two decades to technically improve such methodologies and/or to overcome the oxidative-induced damage during the processing of embryos. We recently found that culture of rabbit embryos with antioxidant materials such as melatonin [39] and retinol [15] can protect embryos from the oxidative stress and support the *in vitro* development of embryos. In addition, the *in vitro* developmental capacity of vitrified embryos were maintained by pre-culture of embryos with melatonin for 2 h before vitrification [7]. Many studies also indicated that leptin could be used to promote the *in vitro* development of embryos; however, these studies have been focused on the morphological aspects while other aspects and protective effects of leptin against oxidative stress during *in vitro* and cryopreservation conditions have not been sufficiently explored. Previous studies showed that the half-life of leptin in human and rats was determined in a range of 25–71 min [34,40]. Therefore, in the present study, rabbit embryos at the morulae stage were cultured with different levels of leptin for 2 h and then were allowed for further development as fresh or after vitrification to evaluate the morphological aspects of embryonic development *in vitro* and to explore the expression of some pro-oxidant, antioxidant, apoptotic and developmental biomarkers in the fresh and vitrified rabbit embryos.

Our results demonstrated that the addition of leptin at 20 ng/mL significantly improved the expanding and hatching rates while the higher leptin concentration (100 ng/mL) decreased the expanding and hatching rates compared to the free-leptin group in both fresh and vitrified embryos (Fig 2). These results are consistent with previous studies that reported a concentration-dependent effect of leptin on embryonic development in various species [20,31,32,34]. It was reported that competent blastocysts can release higher leptin molecules in culture media compared to arrested embryos of human [33]. The beneficial effect of leptin on the embryonic development was attributed by many researchers to the increased ICM and TE cell number which is directly correlated with embryo quality and viability [20,31,41]. Irrespective of leptin treatment, the vitrification treatment in the present study significantly decreased the expanding and hatching rates of cultured embryos (57 and 51%, respectively) compared to the fresh embryos (78 and 71%, respectively). These results are comparable with other studies in rabbits. For example, a recent study reported that vitrification of morulae embryos using cryotop or calibrated plastic inoculation loop devices resulted in hatching of 62% of vitrified embryos vs. 95% in fresh embryos [6]. Compared to this study, the lower hatching rates of vitrified embryos in our study may be due to using a carrier (0.25 mL straws) with a high volume and velocity of vitrification. Also, the low rate of hatched embryos in fresh embryos in our study could be attributed to the higher dose of eCG used for does receptivity that can affect the quality of recovered embryos [42]. The low development of embryos after vitrification was also documented in previous studies and was attributed to the damage induced by oxidative stress [7,8,10–14]. In addition, Vajta [38] explained that the number of surviving blastomeres in the devitrified embryos may be insufficient for the re-expansion of the blastocoelic cavity and the consequent developmental events.

Leptin was previously reported as antioxidant and free radical scavenger in a rat model suffer from oxidative stress [43,44]. There is only one study reported a positive effect of leptin on cryopreserved men sperm which was induced by the activation of certain antioxidant enzymes [45]. The results of the present study displayed an increase in the levels of MDA and NO as pro-oxidant biomarkers due to the vitrification treatment, while the TAC, SOD and GPx antioxidant biomarkers were significantly decreased. Similar results have been reported previously in vitrified embryos of rabbit [7]. In contrast, leptin treatment before vitrification maintained the levels of MDA and NO at similar levels to that obtained in the fresh embryos. Furthermore, leptin promoted the activation of antioxidant biomarkers in the fresh embryos, and increased SOD and GPx activity in the vitrified embryos treated with leptin compared to those vitrified embryos without leptin (Fig 3). It was also concluded that leptin resistance in lean swine fetuses negatively affected the antioxidant homeostasis and increased the oxidative stress, contributing to low aspects in the reproductive efficiency and embryo viability [46]. In addition, leptin would independently regulate the transcription of 5’-AMP-activated kinase (AMPK) and nuclear factor-kB (NF-kB) in the mitochondria which, in turn, control the energy metabolism, ROS production and oxidative stress [47]. Furthermore, the antioxidative effects of leptin on embryos could be attributed to the overexpression of the redox-sensitive transcription factor, nuclear factor erythroid 2-related factor 2 (Nrf2), which is the main regulator for the antioxidant enzymes system [48,49]. We did not determine Nrf2 in the present study; nevertheless, it was concluded by other works that Nrf2 was overexpressed in vitrified rabbit embryos cultured with antioxidant compounds [7]. This transcription factor has an important role to restore the other antioxidant enzymes of embryos to be more tolerant against oxidative stress and more competent for development [50].

Embryos exposed to *in vitro* and vitrification conditions show low capability of blastulation and expanding [16] due to a strong reduction in the inner cell mass [51]. Earlier studies reported that such conditions may switch on/off some developmentally important genes that play a pivotal role in the regular development and expanding of preimplantation embryos [52–54]. In the present study, some pro-apoptotic and developmental biomarkers were also determined for the first time by ELSA assay in our experimental groups. The negative effects of cryopreservation on embryo expression of developmental genes were well documented previously in porcine [55], mouse [56,57] and human [58,59]. Our results also revealed an increase in the apoptotic proteins along with a decrease in the developmentally proteins due to the negative effects of vitrification treatment on rabbit embryos not previously treated with leptin (Fig 3). In contrast, the positive effect of leptin on fresh embryos in the present study could be attributed to the antiapoptotic action of leptin via downregulation of P53 and MAP kinase pathways which consequently reduce genetic expression of apoptotic proteins like Caspase3, TNFα and BAX [43,60,61]. Furthermore, culture of embryos with leptin before vitrification ameliorated the reduction in the developmental biomarkers induced by the vitrification in the present study (Fig 4B). Such corrective effect of leptin was also demonstrated against apoptosis incidence in hepatocytes of oxidative-injured liver [44]. Moreover, Zheng et al [62] reported that antiapoptotic effect of leptin on cardiomyocytes could be triggered by increasing the antioxidant defense, particularly the SOD activity.

It is known that HSP60 may assist the polypeptides of mitochondrial matrix for refolding and proper assembly under stress conditions [63]. In contrast, it is also suggested that HSP60 release in the extra-mitochondrial cytosol may regulate apoptosis of intact cells through binding to BAX and accelerating caspase-3 activation [64,65]. Results in the current study indicate that culture of embryos with leptin before vitrification significantly increased the level of HSP60 compared to the other groups. This result may support a hypothesis that leptin can restore embryos under cryopreservation conditions to be more tolerant to the oxidative stress through more expression of HSP60 [66]. The increased HSP60 in these embryos may, in turn, facilitate the proper folding of imported mitochondrial proteins and prevent their denaturation [67].

In conclusion, the addition of leptin at 20 ng/mL to the culture media improved the *in vitro* development rates including expanding and hatching rates of fresh and vitrified rabbit embryos. Leptin seems to act directly via the activation of the antioxidant system of embryos. While vitrification induces high levels of apoptotic BAX, HSP60 and TNFα biomarkers in embryos, the pre-culture of embryos with leptin has antiapoptotic effect on the vitrified embryos, and moreover, ameliorates the reduction of the developmental biomarkers such as SOX2, NANOG and OCT4 proteins in the vitrified embryos. Furthermore, leptin may protect the vitrified embryos against oxidative stress via promoting the HSP60 biomarker to support the mitochondrial function. These results indicate that leptin could be used as antiapoptotic and antioxidant promotor to support the development of cultured rabbit embryos under *in vitro* study conditions. Further *in vivo* studies are needed to confirm the positive effects of leptin on the embryonic development when cryopreservation programs are implemented in rabbit species.

## Supporting information

S1 Fig Developmental stages obtained from cultured rabbit embryos in the current study. (A) Compacted morulae embryos recovered from rabbit does 70 h post insemination. (B) Examples of expanding blastocysts obtained after 24 h of embryo culture. (C, D, E and F) Hatched/hatching blastocysts obtained after 48 h of culture in the fresh embryo group without leptin, vitrified embryo group without leptin, fresh embryo group with 20 ng/mL leptin and vitrified embryo group with 20 ng/mL leptin, respectively. Images were captured using a digital video camera (Cohu, Inc., San Diego, CA, USA) connected to a stereo zoom microscope at magnification of 45X (scale bar: 100 μm).
(TIF)
