# Supplementary figures and images for: Leptin improves the in vitro development of preimplantation rabbit embryos under oxidative stress of cryopreservation
Source: PLoS One. 2021 Feb 2;16(2):e0246307. doi: 10.1371/journal.pone.0246307 (PMC7853501; doi:10.1371/journal.pone.0246307)

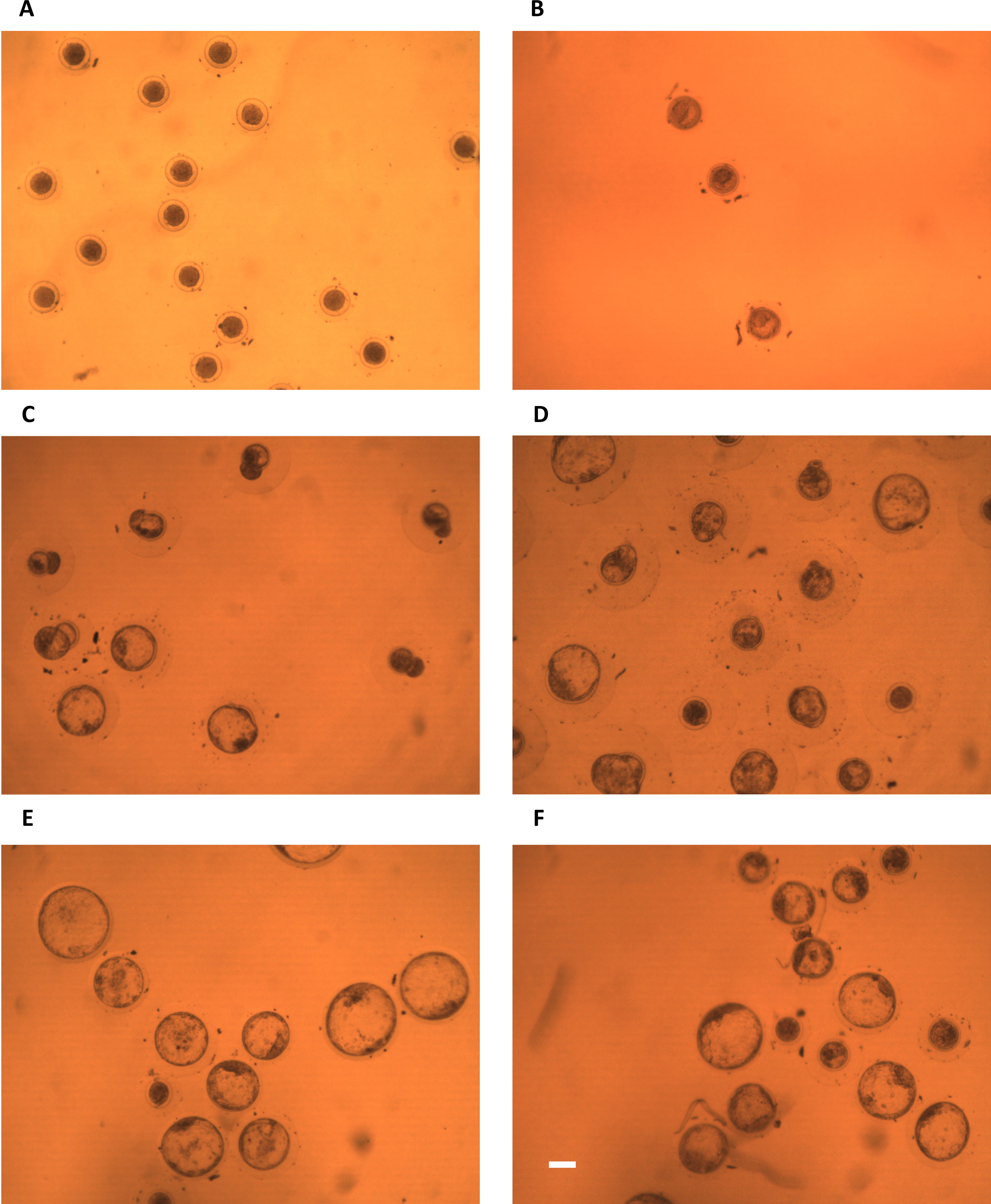

Supplement: S1 Fig — (A) Compacted morulae embryos recovered from rabbit does 70 h post insemination. (B) Examples of expanding blastocysts obtained after 24 h of embryo culture. (C, D, E and F) Hatched/hatching blastocysts obtained after 48 h of culture in the fresh embryo group without leptin, vitrified embryo group without leptin, fresh embryo group with 20 ng/mL leptin and vitrified embryo group with 20 ng/mL leptin, respectively. Images were captured using a digital video camera (Cohu, Inc., San Diego, CA, USA) connected to a stereo zoom microscope at magnification of 45X (scale bar: 100 μm). (TIF) [file pone.0246307.s001.tif]
